# General practitioner views about discussing sexual issues with patients with coronary heart disease: a national survey in Ireland

**DOI:** 10.1186/1471-2296-11-40

**Published:** 2010-05-25

**Authors:** Molly Byrne, Sally Doherty, Hannah M McGee, Andrew W Murphy

**Affiliations:** 1 School of Psychology, NUI, Galway, Ireland; 2 Department of Psychology, Royal College of Surgeons in Ireland, Dublin, Ireland; 3 Department of General Practice, NUI, Galway, Ireland

## Abstract

**Background:**

Sexual problems are common among people with coronary heart disease and can adversely affect patients' quality of life. GPs are ideally placed to deal with these problems. Research suggests that GPs are reluctant to address sexual problems but little is known about what currently takes place in practice. The aim of this study was to examine GPs' self-reported behaviour and attitudes to discussing sexual problems with people with coronary heart disease.

**Method:**

Design: A cross-sectional survey which administered 230 postal questionnaires to a nationally representative, stratified random sample of GPs in the Republic of Ireland. GPs were asked about current practice, knowledge, awareness and confidence in dealing with sexual problems, barriers to addressing sexual problems, and about improving services in this area.

**Results:**

Responses were available for 61 GPs (27% response rate). Seventy percent of GPs reported that they rarely or never discussed sexual problems with coronary patients. While all GPs believed addressing sexual problems was important, many GPs reported lacking awareness, knowledge and confidence in addressing sexual problems. The main barriers were lack of time, feeling the patient wasn't ready and lack of training in the area. GPs wanted more training and guidelines for practice.

**Conclusions:**

There is currently no standardised protocol for GPs for dealing with sexual problems among coronary patients. Awareness of these issues appears to be low among GPs. Services could be improved by developing practice guidelines for brief, effective actions or assessments, providing training in the area and improving information resources and support services for referral.

## Background

Sex and sexuality are core dimensions of the human experience and important determinants of well-being. However, sexual problems are widespread and can adversely affect mood, quality of life and interpersonal relationships[1]. Issues of sexual satisfaction and dysfunction are especially pertinent for health professionals at this time as people now have higher expectations about being able to achieve and sustain satisfying sexual relationships into older age[2].

Among those with cardiovascular disease, sexual dysfunction is highly prevalent in both sexes and has been shown to adversely affect patients' quality of life and well-being[3]. Most patients who develop chronic cardiovascular disease experience an associated decrease in libido and frequency of sexual activity[4] and a significant proportion of men experience erectile dysfunction[5]. Although the majority of the literature in this area deals with male sexual functioning, the available literature suggests that the reduction of frequency and satisfaction with sexual activity following a myocardial infarction is similar for both men and women[6].

In addition, sexual problems may arise for patients with coronary heart disease as patients and their partners may fear a cardiovascular event or sudden death during sexual activity, as normal signs of sexual arousal such as increased heart rate, respiration and sweating can easily be misinterpreted as cardiac symptoms[7]. In fact, the incidence of having a heart attack or life threatening arrhythmia during sexual activity is low[4]. Patients may report feeling anxious about decreased sexual performance after a heart attack or coronary surgery[8]. Anxiety and depression, common in patients after a heart attack, may also have the effect of decreasing libido[9]. In addition, prescribed cardiac medications often have sexual side effects, and may decrease libido or sexual performance[10]. Beta-blockers and lipid lowering drugs have been shown to exacerbate erectile dysfunction in men[4,5]. If sexual problems are not addressed as part of the overall medical care of patients with coronary heart disease, then patients may assume that nothing can be done and may live with major and unavoidable restrictions to their quality of life, and to that of their partner.

The literature suggests that health professionals are frequently reluctant to address this personal aspect of patients' lives[11]. Most cardiologists, nurses and general practitioners do not routinely ask cardiac patients about sexual problems, and patients are often reluctant or embarrassed to mention sex[5]. Even among clinicians who acknowledge the relevance of addressing sexual issues in their patients, there is a general lack of understanding of the optimal approach for sexual problem identification and recognition, while cardiac rehabilitation programmes typically fail to acknowledge the importance of sexual dysfunction concerns in their patients[12]. Research suggests that both male and female patients with coronary heart disease expressed regret that their general practitioner, cardiologist, or surgeon had not broached the subject of sexual function before or after an acute coronary illness episode[13]. In a Swedish study exploring sexual health management among 33 women with type II diabetes, participants reported feeling uncomfortable raising sexual issues with their doctors, although some would have liked to, and only one patient had had sexual health issues raised with her by her doctor[14].

Barriers to providing sexual counselling as identified by health professionals include too little time, lack of knowledge or training, negative attitudes and beliefs about sexuality, a perception that it is someone else's job, patient's lack of readiness, sexuality not seen as a problem by health practitioners, patients perceived as too ill to address sexual issues, concerns about increasing patient anxiety and discomfort in discussing the topic and views about the inappropriateness of sex in later life[2,15]. There also appear to be a number of barriers which prevent patients from initiating discussions, including a perception that individual practitioners do not appear to be experienced or mature enough to understand the patient's problems or feelings of shyness and embarrassment. Also, age and gender issues may discourage the patient from expressing their concerns[16]. The busy setting of acute hospitals, and the increasingly rapid throughput of patients with cardiac conditions and procedures, is another challenge to 'windows of opportunity' for discussing sexual concerns.

General practitioners are ideally placed to initiate and provide continuity on discussions of sexual health issues with people with coronary heart disease[17]. Indeed, GPs seem to be the preferred source of professional help for sexual problems[18]. However, little is known about GPs' experiences of, and attitudes about, discussing sexual health issues with people with coronary heart disease. This study aims to address this gap by surveying a random sample of GPs in the Republic of Ireland.

## Methods

### Design of study

Cross-sectional questionnaire survey set in the Republic of Ireland.

### Participants

The sampling frame was all GPs (n = 2300) listed in the Irish Medical Directory 2008-2009 http://www.imd.ie/. Randomisation was conducted by identifying ten percent of GPs in each county, by noting every tenth GP in each county listed in the Directory. A total of 230 questionnaires were sent to those identified by this selection procedure.

### Study questionnaire

The study questionnaire was developed based on a review of the literature in the area, discussions among the research steering committee and feedback on a draft version from 5 GPs. A copy of the questionnaire can be viewed in additional file 1. The questionnaire comprised 15 mostly closed option questions focusing on background and training characteristics of the GPs and on their experience and views concerning sexual problems in general practice. Sexual problem questions focused on:

(A) Frequency of reports of sexual problems from patients with coronary heart disease during consultations;

(B) The GP's level of knowledge and awareness of, and confidence in dealing with, sexual problems with patients;

(C) Their current practice in sexual assessment and counselling for patients with coronary heart disease and whether they currently adhere to particular guidelines for assessment and counselling;

(D) Perceived barriers to addressing sexual issues in practice. A list of 17 barriers identified in a previous study [19] were listed and GPs were asked to rate their agreement with each barrier for them personally on a 6 point likert scale. Additional barriers could be noted.

(E) Views on management: an open-ended section asked for views about how sexual problems should ideally be managed within primary care and views about the best way forward with regard to service provision in this area.

The questionnaire was piloted with 5 GPs and small changes were made to the wording of some of the questions.

### Procedure

Selected GPs were sent a letter inviting them to participate, the study questionnaire, a study information sheet and a stamped addressed reply envelope. An opt-out card was also provided, which GPs could return if they did not want to be involved in, or further reminded about, the survey. Reminder letters were posted to non-respondents three weeks after the initial mailing.

### Data analysis

Quantitative data were analysed by SPSS (v15). Descriptive statistics were used to estimate the responses while both parametric and non-parametric analyses explored gender differences. Responses to the final open-ended question regarding how sexual problems *should *be managed within primary care were transcribed and analysed qualitatively as follows. Two members of the research team (SD and MB) independently read each response and sorted responses into basic categories, based on perceived similarities and differences. The summary lists of categories generated by each researcher were compared. Differences were discussed and categories were refined. A final comprehensive list was prepared.

### Ethical approval

The study was granted ethical approval by the National University of Ireland, Galway Research Ethics Committee in June 2008.

## Results

Sixty-one GPs (40 males and 21 females) completed the questionnaire (27% response rate). Forty-two GPs (23 males and 19 females) returned their card opting out of the study and 126 GPs (88 males and 38 females) did not respond. A Chi-squared test of independence indicated there was no significant difference between gender of respondents and those who did not respond (χ^2 ^(1, n = 230) = 0.95; p = 0.82.). Sixty-six percent (n = 40) of the respondents were male and the mean age of the overall sample was 50 years (SD = 8.8 years). Nearly half the GPs (n = 28, 46%) worked in urban practices, 12 (20%) worked in rural practices and 21 (34%) worked in mixed urban/rural practices.

The frequency of discussions which GPs reported about sexual health problems with patients with coronary heart disease is summarised in Table 1. Overall, the majority of GPs report that they never or rarely address sexual health problems with patients with coronary heart disease, either in the early stages post diagnosis (70%) or among patients with more long term diagnoses (64%). However, GPs seem slightly more inclined to discuss sexual health problems with patients with a less recent diagnosis, with 34% of GPs reporting that they sometimes discuss sex with patients who have been diagnosed more than 3 months previously. Eighty-four percent of GPs reported newly diagnosed patients rarely or never report sexual health problems; this figure drops to 71% of patients who have been diagnosed more than 3 months previously.

**Table 1:** GPs' reports of frequency of different aspects of discussions of sexual health problems during consultations with patients with coronary heart disease.

***Aspect of discussing sexual health problem***	***Always***	***Frequently***	***Sometimes***	***Rarely***	***Never***
Frequency of discussing sexual health problems with newly diagnosed patients (<3 months)	2	2	26	54	16
Frequency of discussing sexual health problems with patients *after *3 months post diagnosis	0	2	34	51	13
Frequency of newly diagnosed patients (<3 months) reporting sexual health problems	0	0	16	72	12
Frequency of patients more than 3 months post diagnosis reporting sexual health problems	0	1	28	61	10

Note: percentages are reported, N = 61.

Sixty-nine percent of GPs (n = 42) reported that, in general, the patient is more likely than the GP to initiate conversations around sexual health problems. When asked about their own perceived importance of discussing sexual health problems with patients, all GPs believed it was of some importance: just over half of GPs (57%) believed that it was 'somewhat important', 38% believed it was 'very important' and 5% believed it was 'extremely important'. GPs reported that these issues were also important for their patients; 46% believed that they were 'somewhat important' to patients, 44% believed they were 'very important' and 10% believed they were 'extremely important'.

When asked to rate their knowledge, awareness and confidence in dealing with sexual health problems, GPs generally perceived themselves as fair/good, with few rating themselves as either poor or very good/excellent. The breakdown of these ratings is shown in table 2.

**Table 2:** GPs' ratings of themselves in terms of knowledge, awareness and confidence in dealing with sexual health problems during consultations with patients with coronary heart disease.

***Competency***	***Excellent***	***Very good***	***Good***	***Fair***	***Poor***
Knowledge	0	15	62	21	2
Awareness	2	10	39	41	8
Confidence	1	20	41	31	7

Note: percentages are reported, N = 61.

When asked about whether they followed specific guidelines in dealing with sexual health problems among this patient group, no GP reported following specific guidelines in assessing sexual health problems and only one GP (out of 61) reported following specific guidelines for counselling people with sexual health problems. This GP reported following guidelines learned during training as a psycho-sexual therapist.

A minority of GPs (n = 7, 11%) said that in the past they had referred patients with coronary heart disease onto other services for sexual health problems. GPs reported referral to a counsellor (n = 4), a sex therapist (n = 2) or an urologist (commonly for erectile dysfunction, n = 3).

Agreement with listed barriers reported as preventing GPs from discussing sexual health problems is documented in table 3. An independent t-test was conducted to compare total barrier scores for male GPs and female GPs. There was no significant difference for males (m = 50.9, SD = 12 and females m = 52.4, SD = 11.7; t (58) = -.427, p = .639, 95% CI: -8.02 to 4.9) Fourteen GPs wrote in an open ended section additional barriers which were not listed on the barrier menu. The most commonly stated additional barrier was lack of awareness, which was a stated barrier for 6 GPs. Other barriers included patients having difficulty discussing sexual issues, not knowing when is the 'right time' to raise sexual issues, patients being reluctant to discuss sexual issues as they have illegally bought Viagra, a difficulty raising sexual issues in a sensitive way, public patients being unable to pay for private sex therapy services and a belief that it is an inappropriate topic for some patients.

**Table 3:** Number and percentage of GPs reporting agreement (those who ticked partly agree, agree or strongly agree) and average agreement score (1 = strongly disagree; 6 = strongly agree) with listed barriers to discussing sexual health problems with patients with coronary heart disease.

***Barrier***	***n agreeing***	***% agreeing***	***Mean agreement score (1-6)***
Not enough time	44	72	4.0
Patient's lack of readiness	44	72	3.9
Lack of training	38	62	3.7
Concerns about increasing patients' anxiety and discomfort	33	54	3.5
Patients perceived as too ill to address sexual issues	31	51	3.5
Sexuality not seen as a problem by patient	30	50	3.4
Elderly age of patient	30	49	3.4
Presence of a third party	28	46	3.4
Fear of offending the patient	25	41	3.2
Lack of knowledge	19	31	3.0
Issues relating to language and ethnicity	17	28	2.7
Patient of opposite sex to GP	17	28	2.6
Issues relating to culture and religion	16	26	2.8
Embarrassment	12	20	2.5
GP's own negative attitudes and beliefs about sexuality	8	13	2.0
Perception that it is someone else's job	5	8	1.9
A large age difference between GP and the patient	4	6	2.0

(N = 61)

Seventy percent (n = 43) of GPs provided a response to the question: 'How do you think sexual problems should ideally be managed within primary care for patients with coronary heart disease?' Responses were organised into 8 categories, which are listed, with illustrative quotations, in table 4.

**Table 4:** Categories of responses to the question: 'How do you think sexual problems should ideally be managed within primary care for patients with coronary heart disease?'

*Category*	*Illustrative quotation*
More training/education needed for GPs	*'The GP is the ideal first port of call for such issues, but we do need help in learning how to raise and deal with such issues'. GP030*
GPs need to raise the issue during consultations	*'We should bring it [sex] up....bringing it up may not reveal any problems at the time, but gives patients an opportunity to come back to it at a later date'. GP035*
GPs need to provide the time and opportunity during consultations for patients to raise sexual issues	*'Frequently in primary care when the subject is approached there can be a negative response from the patient - as if their privacy has been encroached upon - on occasions, a patient would leave the practice due to this incursion. Thus, if I seek an answer I will try to get the patient to initiate the discussion by discussing bodily function and effects of physical and emotional disturbance'. GP076*
Guidelines/protocols need to be developed to guide GP behaviour	*'A protocol for management ought to be developed for use by GPs'. GP052. 'If we are to raise the issue then some evidence-based guidelines would be helpful to guide management'. GP064*
Hospital cardiac rehabilitation should play central role	*'[Sexual issues] should be addressed specifically as part of hospital cardiac rehabilitation program at presentation'. GP131*
Needs to be more awareness among GPs	*'Erectile dysfunction is currently being highlighted - we must continue to raise awareness'. GP131*
Resources should be developed, e.g. leaflets/DVDs to give to patients during consultations	*'There should be patient information leaflets to give patients with coronary heart disease that discuss the sexual problems they may encounter. This would open up the issue for discussion'. GP225*
Need other staff/services for referral	*'If such issues are raised though we need to have some confidence in the availability of backup should we get out of our depth. Lack of such backup is also a very big issue.' GP030*

Note: Seventy percent (n = 43) of GPs provided a response to the question.

## Discussion

The results of this study support findings from previous studies that GPs infrequently discuss sexual issues with their patients with coronary heart disease[11]. In addition, only very few GPs had referred patients onto other professionals for sexual problems. Therefore, it is likely that a significant proportion of patients are not discussing sexual problems with any health professional. Indeed, research suggests that GPs are reluctant to discuss sexual issues with patients *in general*[19]. Previous studies have found that most cardiologists, nurses and primary care physicians do not routinely ask cardiac patients about sexual problems [5,13]. The current study suggests that most GPs acknowledge that it *is *important to discuss sexual issues with their patients and they also believe that these issues are important ones for their patients. However, in line with previous research[12], there is a general lack of agreement regarding the optimal approach for sexual problem identification and recognition. When asked in this study to rate their own knowledge, awareness and confidence in dealing with sexual issues in practice, GPs tended to rate themselves at the middle to lower end of the scale. This was particularly prominent for 'awareness', where around half of the sample claim their awareness of sexual problems among this particular group of patients was either poor or fair.

As in previous studies[2,15], the most commonly reported barrier to discussing sexual problems with patients is a lack of time. In the current study, a perception that the patient is not ready to discuss sexual problems and a perceived lack of GP training in sexual problem management were also significant barriers. Concerns about increasing patient anxiety and discomfort, a perception that patients are too ill to address sexual issues and a perception that patients don't see sexual problems as an issue were also endorsed by the majority of GPs. A lack of awareness of sexual issues was raised by a significant number of GPs. Interestingly, lack of knowledge, being of opposite sex to the patient or embarrassment, which had been found to be barriers in previous research[2,15], do not appear to be barriers for the majority of GPs in the current study.

Most GPs who responded to this survey were keen to give a view about how sexual problems should ideally be managed in general practice. A majority believe that further training and education are required and that there is a need for formal guidelines and protocols to be developed. GPs suggested that developing resources such as leaflets and/or DVDs dealing with sexual problems would be a useful resource to give to their patients. GPs believe that it is very important to raise sexual issues during consultations, or at least, provide the opportunity for patients to raise such issues if they are relevant to them. While most GPs agree that primary care has a central role in providing advice and support about sexual problems, a number of GPs highlighted that hospital cardiac rehabilitation also has an important role. The issue should at least be raised during hospital rehabilitation, which may prompt patients to discuss such issues later with their GPs. Finally, GPs highlighted that there should be greater availability of specialist services for more serious cases of sexual dysfunction, to which they can refer patients where the need arises.

In the majority of instances where discussions about sex took place, it seems the patient is the one who initiates the discussion. In open-ended sections of the questionnaire, GPs indicated that they were reluctant to raise sexual issues for fear of offending the patient. However, evidence suggests that Irish people *are *increasingly willing to discuss sexual issues with their health care practitioner: the Irish Study of Sexual Health and Relationships[20], a survey of sexual issues among a general population sample, found that over half of men (52%) and women (58%) reported that it would be easy or very easy for them to discuss a sexual problem with a health professional. Furthermore, of the small number who had already spoken about such problems, the majority had spoken to a GP (67% of men and 46% of women). The provision of leaflets/DVDs dealing with sexual problems, as suggested by GPs in the study, may be a particularly useful strategy to enable GPs to raise sexual issues in a non-threatening way, opening channels of communication for both GP and patient.

### Strengths and limitations of the study

Little research has taken place to date on the topic of sexual issues and how they are dealt with among patients with coronary heart disease in general practice; therefore the current study is novel. Indeed, this study found that a significant number of GPs admitted to having low levels of awareness of this problem when completing the questionnaire. In an endeavour to maximise our response rate our questionnaire was short and concise, and we paid significant attention to questionnaire planning and design. We were rewarded for this effort with minimal missing data among returned questionnaires.

However, caution needs to be applied when interpreting the results of this study. The findings may be affected by non-response bias, given the low response rate (27%). Low response rates are a common limitation of research in general practice and evidence suggests that it is becoming increasingly more difficult to encourage GPs to participate in surveys as they receive more and more requests to do so [21]. Though low, the response rate in the current study is comparable to similar Irish surveys in general practice reported in the literature [22]. In addition, the profile of characteristics for GPs responding to this survey (in terms of gender, age and rural/urban setting of practice) is similar to that reported for GPs nationally in a 2005 survey [23,24]. A t-test comparison revealed no significant differences for gender and total barrier scores; this may well be as a result of a type two error, due to small size. Non-responders in general are more likely to be those who are uninterested in or dislike the topic being researched or those who may be threatened by the nature of the research topic[25]. Therefore, if anything, the current study is likely to present a more optimistic picture than actually exists with regard to current practice and attitudes towards sexual counselling in general practice. In the 'post-Viagra' world of increasing publicity concerning erectile dysfunction, it was important to assess the experience of contemporary GPs - what doctors and their patients are doing - albeit as reported by GPs only in this study.

## Conclusions

Among those with cardiovascular disease, sexual dysfunction is highly prevalent in both sexes and has been shown to adversely affect patients' quality of life and well-being [3]. If sexual problems are not addressed as part of the overall medical care of patients with CHD, then patients may assume that nothing can be done and may live with major and unavoidable restrictions to their quality of life, and to that of their partner. GPs in the current study reported that they rarely asked patients with coronary heart disease about sexual problems and indicated that in general, they had only low levels of awareness of sexual problems among this patient group. The findings from this study suggest that there is a need to increase awareness of this issue among GPs. GPs themselves also indicated that they would like access to more specific guidelines for practice and would value further training in this area. As one of the main barriers noted to addressing sexual problems in practice was a lack of time, it is also important that training initiatives address the challenge of developing brief, effective actions or assessments delivered by GPs, alongside more specialist ancillary services for sexual counselling where patients with more challenging or complex sexual problems can be referred. Future research should focus on developing and evaluating interventions to increase and improve sexual function assessment and counselling in general practice.

## Abbreviations

GP: General Practitioner; CHD: Coronary Heart Disease; SPSS: Statistical Package for the Social Sciences.

## Competing interests

The authors declare that they have no competing interests.

## Authors' contributions

MB conceived the study, and participated in its design and co-ordination and drafted the manuscript. SD participated in the design and co-ordination of the study, collected and prepared study data and helped to draft the manuscript. HMcG participated in the design and co-ordination of the study and helped to draft the manuscript. AWM participated in the design and co-ordination of the study and helped to draft the manuscript. All authors read and approved the final version.

## Pre-publication history

The pre-publication history for this paper can be accessed here:

http://www.biomedcentral.com/1471-2296/11/40/prepub

## Supplementary Material

Additional file 1 **Study Questionnaire**. The file contains a copy of the questionnaire sent to GPs. Click here for file
