# Supplementary material for: General practitioner views about discussing sexual issues with patients with coronary heart disease: a national survey in Ireland
Source: BMC Fam Pract. 2010 May 25;11:40. doi: 10.1186/1471-2296-11-40 (PMC2886005; doi:10.1186/1471-2296-11-40)
Supplement: Additional file 1 — Study Questionnaire. The file contains a copy of the questionnaire sent to GPs. [file 1471-2296-11-40-S1.PDF]

OÉ Gaillimh
NUI Galway

RCSI

The *CHARMS* Study

Cardiac Health and Assessment of Relationship Management and Sexuality

Funded by the Health Research Board

<u>National Survey of General Practitioners</u>

Current practice of sexual assessment and management of patients with coronary heart disease in primary care in Ireland

This survey is the first phase of a three phase study, in which we aim to develop recommendations for how services should be developed to meet the needs of coronary heart disease patients with sexual health problems.

We are also contacting all cardiac rehabilitation services through out the country with a similar survey.

In Phase Two we will speak to the patients to get their views.

In Phase Three of the study, a small sample of GPs, cardiac rehabilitation staff and patients will be asked to take part in a number of group interviews to give their views and to discuss the findings from Phase One and Two.

All of the information you provide will be treated with confidentiality and only the researcher will have access to this information.

If you are interested in taking part in this survey please complete the questionnaire and return it in the freepost envelope provided.

This survey should take no more than 15 minutes to complete.

Thank you for your help

Section One: Details about you and your practice

Please provide your personal details:

1.0 Male ☐ **Female** ☐

1.2 Please state your age ☐ **Years**

1.3 Year of qualification in Medicine ☐

1.4 How many doctors, including yourself, currently work in your practice?

(a) Full time ☐ **(b) Part time** ☐

1.5 Location of your practice

(a) Urban ☐ **(b) Rural** ☐ **(c) Mixed** ☐

1.6 Region of your practice: please insert your telephone code (e.g. 091) ☐

1.7 Approximately how many patients <u>overall</u> are currently in your practice? ☐

1.8 Approximately how many GMS patients do you currently have in your practice? ☐

1.9 Are you part of the ICGP Heartwatch scheme? **YES** ☐ **NO** ☐

Section Two: Sexual health problems after coronary heart disease

2. How often do <u>you</u> discuss sexual health problems with newly diagnosed (less than 3 months) coronary heart disease patients during consultations?
Please tick one √

Always	Frequently	Sometimes	Rarely	Never

3. How often do <u>you</u> discuss sexual health problems with coronary heart disease patients after this 3 month period during consultations?
Please tick one √

Always	Frequently	Sometimes	Rarely	Never

4. How often do newly diagnosed (less than 3 months) <u>patients</u> with coronary heart disease report sexual health problems during consultations?
Please tick one √

Always	Frequently	Sometimes	Rarely	Never

5. How often do coronary heart disease patients after this 3 month period report sexual health problems?
Please tick one √

Always	Frequently	Sometimes	Rarely	Never

6. If these discussions do take place who is most likely to initiate the conversation around sexual health problems after coronary heart disease
Please tick one √

(a) You ☐ **(b) The Patient** ☐

7. In your view how important do you think it is for the GP to discuss these issues with their patients?
Please tick one√)

Extremely important	Very important	Somewhat important	Not important

8. In your view how important do you think these issues are for your patients?
Please tick one√

Extremely important	Very important	Somewhat important	Not important

9. How would you rate your knowledge, awareness and confidence in dealing with sexual problems with patients who have coronary heart disease?
Please tick one for each √

	Excellent	Very good	Good	Fair	Poor
Knowledge					
Awareness					
Confidence					

10. Are there current specific guidelines which you follow for <u>ASSESSMENT</u> of sexual health problems for patients with coronary heart disease? Please tick one√

YES ☐ **OR** **NO** ☐

If YES please give details

____
____
____
____
____
____

11. Are there current specific guidelines which you follow for <u>COUNSELLING</u> sexual health problems for patients with coronary heart disease? Please tick one√

YES ☐ **OR** **NO** ☐

If YES please give details

____
____
____
____
____
____

12. Have you in the <u>PAST</u> referred patients onto other services for sexual health problems related to coronary heart disease?

YES ☐ **OR** **NO** ☐

If YES please indicate which of the following services you may have referred patients to… (Tick as many as apply)

Counselling ☐ **Sex therapist** ☐

Marriage guidance counsellor ☐ **Psychologist** ☐

Other (please specify)

____

____

____

____

13. Below is a possible list of barriers which may <u>prevent you</u> from discussing sexual health problems with coronary heart disease patients. To what extent would you Strongly Disagree (SD) Disagree (D), Probably Disagree (PD) or Probably agree (PA) Agree (A) Strongly Agree (SA) with these statements? Please <u>circle one number</u> on each line.

	DISAGREE SD	D	PD	PA	AGREE A	SA
**Not enough time**	1	2	3	4	5	6
**Lack of knowledge**	1	2	3	4	5	6
**Lack of training**	1	2	3	4	5	6
**My own attitudes and beliefs about sexuality**	1	2	3	4	5	6
**Perception that is someone else's job**	1	2	3	4	5	6
**Patient's lack of readiness**	1	2	3	4	5	6
**Sexuality not seen as a problem by the patient**	1	2	3	4	5	6
**Patients perceived as too ill to address sexual issues**	1	2	3	4	5	6
**Concerns about increasing patients anxiety and discomfort**	1	2	3	4	5	6
**Issues relating to culture and religion**	1	2	3	4	5	6
**Issues relating to language and ethnicity**	1	2	3	4	5	6
**Too large an age difference between you and the patient**	1	2	3	4	5	6
**Patient of opposite sex to you**	1	2	3	4	5	6
**Presence of a third party**	1	2	3	4	5	6
**Embarrassment**	1	2	3	4	5	6
**Fear of offending the patient**	1	2	3	4	5	6
**Elderly age of patient**	1	2	3	4	5	6

14. Are there any other barriers not mentioned above?

YES ☐ **OR** **NO** ☐

If YES please give details

____

____

____

____

____

____

15. We are interested in your views. How do you think sexual problems should ideally be managed within primary care for patients with coronary heart disease?

Thank you for your participation in this survey. If you require any further information please contact:

Sally Doherty 091495954 or email sally.doherty@nuigalway.ie

PLEASE RETURN THE COMPLETED QUESTIONNAIRE IN THE PREPAID ENVELOPE PROVIDED
